# Hospital characteristics associated with highly automated and usable clinical information systems in Texas, United States

**DOI:** 10.1186/1472-6947-8-39

**Published:** 2008-09-15

**Authors:** Ruben Amarasingham, Marie Diener-West, Laura Plantinga, Aaron C Cunningham, Darrell J Gaskin, Neil R Powe

**Affiliations:** 1 Department of Medicine, UT Southwestern Medical Center and Parkland Health & Hospital System, Dallas, USA; 2 Department of Biostatistics, Bloomberg School of Public Health, Johns Hopkins University, Baltimore, USA; 3 Department of Epidemiology, Johns Hopkins Bloomberg School of Public Health and Welch Center for Prevention, Epidemiology and Clinical Research, Baltimore, USA; 4 Department of Data Management and Clinical Research, Parkland Health and Hospital System, Dallas, USA; 5 Department of African American Studies, University of Maryland, College Park, USA; 6 Department of Medicine, Johns Hopkins University School of Medicine, Departments of Epidemiology and Health Policy & Management, Bloomberg School of Public Health and Welch Center for Prevention, Epidemiology and Clinical Research, Johns Hopkins University School of Medicine, Baltimore, MD, USA

## Abstract

**Background:**

A hospital's clinical information system may require a specific environment in which to flourish. This environment is not yet well defined. We examined whether specific hospital characteristics are associated with highly automated and usable clinical information systems.

**Methods:**

This was a cross-sectional survey of 125 urban hospitals in Texas, United States using the Clinical Information Technology Assessment Tool (CITAT), which measures a hospital's level of automation based on physician interactions with the information system. Physician responses were used to calculate a series of CITAT scores: automation and usability scores, four automation sub-domain scores, and an overall clinical information technology (CIT) score. A multivariable regression analysis was used to examine the relation between hospital characteristics and CITAT scores.

**Results:**

We received a sufficient number of physician responses at 69 hospitals (55% response rate). Teaching hospitals, hospitals with higher IT operating expenses (>$1 million annually), IT capital expenses (>$75,000 annually) and hospitals with larger IT staff (≥ 10 full-time staff) had higher automation scores than hospitals that did not meet these criteria (p < 0.05 in all cases). These findings held after adjustment for bed size, total margin, and ownership (p < 0.05 in all cases). There were few significant associations between the hospital characteristics tested in this study and usability scores.

**Conclusion:**

Academic affiliation and larger IT operating, capital, and staff budgets are associated with more highly automated clinical information systems.

## Background

An emerging evidence base suggests that clinical information technologies, such as electronic medical records, computerized order entry, and electronic decision support, can improve the quality of care within the hospital environment [1,2]. U.S. hospitals are rapidly trying to expand their capabilities in these areas but informaticians have long recognized that effective information systems do not emerge fully formed, Athena-like, from the point of purchase. Examples of failure in design, implementation and planning abound [3-6] and hospital systems, healthcare policy makers, and software developers are interested in how to best design and support these systems for the healthcare environment.

To flourish, an information system may require a specific blend of hospital or organizational characteristics in which to root [7-9]. The precise mix of this "nutrient environment" is not well defined. Attempts to characterize this environment have been challenged by a lack of standardized instruments that measure the degree to which a hospital information system is automated [10,11]. A reliable measurement system must be constructed using a *socio-technical *view of inpatient medicine [12]. This view holds that the delivery and quality of clinical care is influenced by dynamic interactions between the social aspects of an organization, i.e., its policies, norms, and culture, and its technical routines, such as those imposed by an information system [12].

We previously developed a clinical information technology assessment tool (CITAT) that quantitatively measures a hospital's level of automation and usability based on a physician's daily interaction with their information system [13]. The instrument was designed and tested using a socio-technical view of inpatient clinical practice and has demonstrated reliability and validity [13,14]. In this study, we examine the relation between specific organizational characteristics, i.e., the "nutrient environment," and the degree to which the hospital information system is automated and usable, as measured by scores on the CITAT.

We hypothesized that investment in the human resources that support information technologies, such as the size of a hospital's IT staff, would be associated with more usable clinical information systems. We also hypothesized that a hospital's automation score would be positively associated with bed size, ownership, financial strength, and teaching status. Urban hospitals that take care of underserved or minority populations in the United States, often labeled "safety net hospitals," frequently have fewer financial resources at their disposal. Some authors have suggested that current disparities in health care may be perpetuated if such hospitals are not assisted in the movement to digitization [11]. We hypothesized that urban safety net status would be negatively associated with automation and usability.

## Methods

### Study Design and Study Population

We conducted a cross-sectional study of urban hospitals in the state of Texas. We chose Texas because it contains among the largest number and variety of hospital organizations in the United States, several different metropolitan areas, and diverse physician and patient populations. We sampled from 125 general, acute care hospitals located within 10 geographically dispersed metropolitan statistical areas (MSAs) in Texas (Abilene, Austin, Dallas, El Paso, Houston, Laredo, Lubbock, McAllen, San Angelo, and San Antonio). We excluded rural, pediatric, specialty, or long-term care facilities or hospitals that were in the process of closing or merging with another facility. The Johns Hopkins University School of Medicine Institutional Review Board approved our research protocol.

### Dependent Variables

The physician-based clinical information technology assessment tool was produced in eight steps according to established methods of survey development. These steps included: development of a conceptual model, literature review, content identification, item construction, pre-testing, item selection, and item re-classification. The CITAT instrument was further tested and validated in four diverse U.S. hospitals, and demonstrated discriminant validity, convergent validity, reliability, and precision [13]. The instrument received subsequent testing in a study of intensive care unit information systems [14].

The CITAT assesses a system's *automation *and *usability*. *Automation *represents the degree to which clinical information processes in the hospital are fully computerized and is divided into four distinct sub-domains: *test results*, *notes & records*, *order entry*, and a set of other sub processes largely consisting of *decision support *[13]. To score highly on a given automation sub-domain, the CITAT requires three factors of routine information practices: 1) the practice must be available as a fully computerized process; 2) the physician must know how to activate the computerized process; and 3) he or she must routinely choose the computerized process over other alternatives, such as writing an order or making a telephone call. *Usability *represents the degree to which information management is effective and well supported from a physician standpoint, regardless of whether a system is automated or manual. An overall measure, called the CIT score, represents an average of the automation and usability scores (the survey items can be obtained from the corresponding author).

Using the American Medical Association (AMA) master file, we selected a 50% random sample of Texas physicians from those who were indicated: 1) to have practice locations in the designated MSAs; and 2) to be practicing internal medicine (including 9 sub-specialties), general surgery (including 10 sub-specialties), or family practice (n = 7,432). We mailed surveys to each of the selected physicians between December 2005 and May 2006. We asked the physician to indicate whether they practiced inpatient medicine, and, if so, to select the hospital in which they provide the majority of their inpatient care. To be eligible, physicians had to actively practice in one of the 125 hospitals selected for this study. As guided by prior work, hospitals for which we did not receive five randomly sampled physician responses were eliminated from further analysis due to the possibility of unstable estimates [14].

The CITAT contains additional items that elicit the background characteristics of the respondents. This included information on the number of inpatient hours provided by the physician in a given week and the number of years practiced at the designated hospital. In addition, computer familiarity and attitude toward computers were assessed through three separate items that were used in previous deployments of the CITAT. Age, sex, specialty, and year of medical school graduation were obtained through the AMA master file. This information was used to assess potential relationships between IT scores and respondent characteristics that might be required for statistical adjustment.

### Independent Variables

Hospital characteristics were obtained from the 2005 survey of the Texas Hospital Association and the American Hospital Association (AHA) annual survey of Texas hospitals. For each hospital in our sample, we obtained the ownership status (public, private/non-profit, and private/for-profit), bed size, total margin, IT operating expense, IT capital expense, and IT staff size. Hospitals were characterized as teaching if they possess a Council of Teaching Hospitals (COTH) status designation. Safety net hospitals were defined using previously established financial classifications [15].

### Statistical Analysis

For each respondent, an automation score, usability score, and four separate sub-domain scores were calculated using methodology previously described [13,14]. Each hospital was then assigned the median value of the scores derived from respondents affiliated with that hospital. Hospital characteristics were dichotomized based on the median value for hospitals in the target sample.

The major objective of our analysis was to examine the relationship between hospital characteristics and CITAT scores, after identifying and adjusting for potential confounders. We first examined whether any responder characteristics such as physician specialty, age, computer orientation, computer sophistication, years of practice at the hospital, or number of hours delivering care at the hospital were independently associated with the dependent variables (CIT, automation or usability) and independent variables (hospital characteristics). Student's *t *test and analysis of variance methods revealed no independent, simultaneous relationships. Thus respondent characteristics were eliminated as potential confounders. This finding was consistent with the results of previous work [13].

We separately compared the mean CIT, automation, and usability scores for each hospital characteristic using either Student's *t *test or analysis of variance. This crude analysis of hospital characteristics was a means to identify potential confounders that would require adjustment in a multivariable regression. We then tested the presence, strength, and independence of associations between each of the hospital characteristics and the CIT, automation, and usability scores using linear regression models, adjusting for the percentage of complete responses and accounting for possible within-hospital clustering of physician responses by using robust variance techniques. Three variables, bed size, ownership, and total margin, were highly correlated with other hospital characteristics (control, operating margin, and debt service coverage) and were also associated with either the automation or usability scores. We included these variables in each of the multi-variable regression models examining the relationship between hospital characteristics and CITAT scores. To normalize the expenditures for IT operating expenses, capital expenses, and IT staff for hospital size, we performed a sensitivity analysis examining the relationship of each of these independent variables, divided by bed size, with the dependent variables. Results were considered statistically significant if p value ≤ 0.05. STATA version 8.2 (College Station, TX) was used for all analyses.

## Results

### Response Rate and Characteristics of Study Hospitals

We received five or more physician responses for 69 of the 125 targeted hospitals (55% response rate). Response rates were generally robust across hospital categories (Table 1); we had excellent response rates among teaching hospitals, safety net hospitals, and hospitals with large IT staffs (83%, 87%, and 74% respectively). Hospitals with smaller bed size, lower total margin, lower operating margin, or lower IT staff had lower response rates (41–45%).

**Table 1:** Characteristics of Responding Hospitals

Hospital Characteristic*	# Responding (%)	Response Rate (%)^∫^
Teaching Status		
Teaching	10 (14%)	83%
Non-teaching	59 (86%)	52%
Urban Safety Net Status		
Urban safety net	7 (10%)	87%
Non-urban safety net	62 (90%)	53%
Bedsize		
<350	39 (57%)	45%
≥ 350	30 (43%)	83%
Ownership		
Not-for-profit	35 (53%)	50%
Government	8 (12%)	70%
For-profit	23 (35%)	44%
Average Age of Plant		
<10 years	45 (66%)	54%
≥ 10 years	23 (34%)	61%
Total Margin		
< 0.03	22 (32%)	44%
≥ 0.03	47 (68%)	65%
IT Operating Expense		
< $1 M	20 (30%)	41%
≥ $1 M	47 (70%)	66%
IT Capital Expense		
< $75,000	24 (41%)	50%
≥ $75,000	35 (59%)	56%
Total IT Staff		
<10	34 (52%)	45%
≥ 10	31 (48%)	74%

We received 5 or more physician responses from 69 hospitals. We did not have hospital characteristic data for all hospitals; thus some categories total less than 69.

In calculating the % response rate for categories where missing values were present, the denominator was reduced by the # of hospitals for which there was a missing value.

Responding physicians were older (average age, 50 years) than non-responding physicians (average age, 47 years). The percentage of participating physicians who were male (81%) was not significantly different than the percentage of male physicians who did not participate (78%). Of all participating physicians, 43% specialized in internal medicine, 36% in surgery, and 22% in family practice. These proportions were similar to those among non-participating physicians. Responding physicians were asked to indicate how many hours a week they spend delivering inpatient care at their hospital. The percentage of respondents who practice <10 hours per week, 11–20 hours per week, and 21–40 hours per week were similar at 24%, 26%, and 20%. Slightly fewer respondents reported working 41–60 hours per week (14%) or > 60 hours per week (16%). As would be expected, the proportion working >40 hours per week was higher among teaching hospitals (47% vs. 24% in non-teaching hospitals) and hospitals with larger bed size (37% vs. 19%).

### Distribution of CITAT Scores

**Figure 1:**
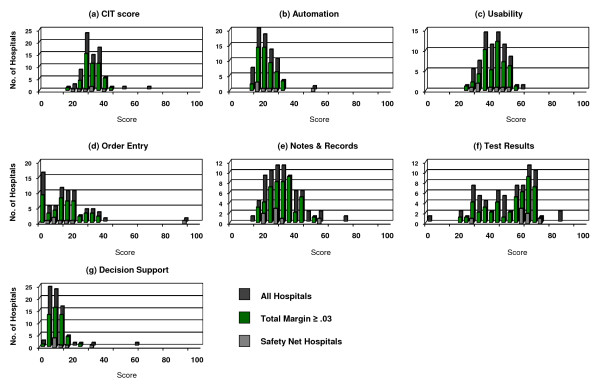
Distribution of CITAT scores: a) CIT; b) automation; c) usability; d) order entry; e) notes & records; f) test results; and g) decision support for all hospitals, hospitals whose total margin exceeds the median for all hospitals (≥ 0.03), and safety net hospitals.

Overall CITAT scores were low in this sample of hospitals (Figure 1). The median automation score was 18.3 (out of a total of 100 points), with a floor at 8.2 points. The usability score was higher than both CIT and automation, with a median score of 40.6. The CIT score, an average of the automation and usability scores, was normally distributed and also exhibited low values (median, 29.1). Most hospitals scored poorly on order entry and decision support, both of which had floors at 0 points and median values of 11.7 and 5.3, respectively. Notes and records and test results had the broadest distribution with higher median values of 28.7 and 53.4, respectively. The median total margin for hospitals in this study was 0.03; both safety net hospitals and hospitals that exceeded a total margin of 0.03 follow the distributions of other hospitals (Figure 1).

### Relationship between Hospital Characteristics and CITAT Scores

CITAT scores were related to several hospital characteristics. In the unadjusted analysis of mean scores, automation scores were statistically significantly greater across multiple hospital characteristics (Tables 2 and 3). Academic hospitals and hospitals with larger bed sizes, IT operating expenses, and IT staff demonstrated higher mean CIT and automation scores than hospitals with fewer beds (p < 0.05 in all cases). Hospitals with higher IT operating expense, IT capital expenses, and larger IT staff had greater mean automation scores (p < 0.05 in all cases).

**Table 2:** Automation, Usability and Clinical Information Technology (CIT) Scores by Hospital Characteristic

		Crude		Unadjusted		Adjusted*	
		
Hospital Characteristic	Physicians, n (%)	Mean	p	β	p	β	p
		Automation					
Teaching Status							
Non-teaching	474 (77%)	18.6		ref	ref	ref	ref
Teaching	141 (23%)	23.6	<.001	4.92	0.089	3.38	0.172
Urban Safety Net Status							
Non-safety set	535 (87%)	19.1		ref	ref	ref	ref
Safety net	80 (13%)	23.6	0.008	4.62	0.311	0.463	0.92
Bedsize							
<350	268 (44%)	18.1		ref	ref	ref	ref
≥ 350	347 (56%)	21.0	0.012	2.65	0.126	1.34	0.377
Ownership							
Not-for-profit	347 (58%)	20.0		ref	ref	ref	ref
Government	80 (13%)	24.0		4.02	0.368	3.86	0.385
For-profit	170 (29%)	18.0	0.007	-2.43	0.130	-2.24	0.201
Average Age of Plant							
> 10 years	367 (60%)	20.2		ref	ref	ref	ref
≥ 10 years	248 (40%)	19.1	0.353	-1.22	0.493	-3.09	0.093
Total Margin							
< 0.01	236 (38%)	20.92		ref	ref	ref	ref
≥ 0.01	379 (62%)	19.0	0.106	-2.05	0.325	-1.50	0.421
IT Operating Expense							
<$1 M	163 (28%)	16.2		ref	ref	ref	ref
≥ $1 M	428 (72%)	20.7	0.001	4.06	0.006	4.11	0.004
IT Capital Expense							
<$75,000	193 (38%)	16.6		ref	ref	ref	ref
≥ $75,000	313 (62%)	21.8	0.001	5.08	0.004	3.24	0.041
Total IT Staff							
<10	248 (44%)	17.2		ref	ref	ref	ref
≥ 10	318 (56%)	21.5	0.001	4.13	0.018	4.75	0.006
		Usability					
Teaching Status							
Non-teaching	474 (77%)	39.8		ref	ref	ref	ref
Teaching	141 (23%)	42.4	0.089	3.24	0.166	9.3	0.509
Urban Safety Net Status							
Non-safety set	535 (87%)	40.7		ref	ref	ref	ref
Safety net	80 (13%)	38.4	0.219	-1.54	0.636	-3.34	0.424
Bedsize							
<350	268 (44%)	39.0		ref	ref	ref	ref
≥ 350	347 (56%)	41.5	0.058	2.24	0.217	2.08	0.249
Ownership							
Not-for-profit	347 (58%)	40.2		ref	ref	ref	ref
Government	80 (13%)	39.2		-.418	0.904	-0.50	0.888
For-profit	170 (29%)	42.2	0.482	1.41	0.486	1.00	0.650
Average Age of Plant							
> 10 years	367 (60%)	40.3		ref	ref	ref	ref
≥ 10 years	248 (40%)	40.6	0.835	-0.22	0.905	-1.41	0.460
Total Margin							
< 0.01	236 (38%)	39.0		ref	ref	ref	ref
≥ 0.01	379 (62%)	41.3	0.070	2.03	0.271	2.12	0.286
IT Operating Expense							
<$1 M	163 (28%)	40.2		ref	ref	ref	ref
≥ $1 M	428 (72%)	40.9	0.606	0.95	0.628	0.59	0.802
IT Capital Expense							
<$75,000	193 (38%)	41.7		ref	ref	ref	ref
≥ $75,000	313 (62%)	40.1	0.282	-1.23	0.490	-0.70	0.726
Total IT Staff							
<10	248 (44%)	39.6		ref	ref	ref	Ref
≥ 10	318 (56%)	41.4	0.184	2.11	0.257	1.53	0.605
		CIT					
Teaching Status							
Non-teaching	474 (77%)	29.2		ref	ref	ref	ref
Teaching	141 (23%)	33.2	<.001	4.14	0.054	4.64	.002
Urban Safety Net Status							
Non-safety set	535 (87%)	30.0		ref	ref	ref	ref
Safety net	80 (13%)	31.3	.3318	1.83	0.618	-1.21	0.660
Bedsize							
<350	268 (44%)	28.6		ref	ref	ref	ref
≥ 350	347 (56%)	31.3	0.004	2.52	0.080	1.76	0.147
Ownership							
Not-for-profit	347 (58%)	30.1		ref	ref	ref	ref
Government	80 (13%)	31.9		2.09	0.572	1.97	0.598
For-profit	170 (29%)	30.1	0.447	-0.48	0.734	-0.62	0.688
Average Age of Plant							
> 10 years	367 (60%)	30.3		ref	ref	ref	ref
≥ 10 years	248 (40%)	29.9	0.736	-0.52	0.717	-1.93	0.224
Total Margin							
< 0.01	236 (38%)	29.9		ref	ref	ref	ref
≥ 0.01	379 (62%)	30.3	0.739	0.17	0.914	0.54	0.340
IT Operating Expense							
<$1 M	163 (28%)	28.2		ref	ref	ref	ref
≥ $1 M	428 (72%)	30.9	0.011	2.61	0.164	1.94	0.309
IT Capital Expense							
<$75,000	193 (38%)	29.2		ref	ref	ref	ref
≥ $75,000	313 (62%)	31.1	0.080	1.10	0.462	0.74	0.630
Total IT Staff							
<10	248 (44%)	28.5		ref	ref	ref	ref
≥ 10	318 (56%)	31.5	0.002	3.12	0.189	2.48	0.197

* The unadjusted and adjusted regression models were adjusted for percentage of complete responses and clustered by hospital. The adjusted models include coefficients adjusted for bed size, total margin, and ownership. ref-reference group.

**Table 3:** Sub-Domain Scores by Hospital Characteristics

		Order		Notes		Tests	
Hospital Characteristic	Physicians	β_adj _*	p	β_adj _*	p	β_adj _*	p
Teaching Status							
Non-teaching	474	ref	ref	ref	ref	ref	ref
Teaching	141	8.73	0.090	-3.09	0.461	10.32	0.044
Urban Safety Net Status							
Non-safety net	535	ref	ref	ref	ref	ref	ref
Urban safety net	80	-0.57	0.939	-0.15	0.976	-2.81	0.629
Bedsize							
<350	268	ref	ref	ref	ref	ref	ref
≥ 350	347	1.57	0.590	1.39	0.551	9.98	0.004
Ownership							
Not-for-profit	347	ref	ref	ref	ref	ref	ref
Government	80	8.84	0.403	0.79	0.878	-2.49	0.729
For-profit	170	-0.05	0.986	1.89	0.502	-20.65	<.001
Average Age of Plant							
< 10 years	367	ref	ref	ref	ref	ref	ref
≥ 10 years	248	-7.78	0.044	0.03	0.992	1.07	0.785
Total Margin							
<0.02	236	ref	ref	ref	ref	ref	ref
≥ 0.02	379	-6.00	0.140	0.75	0.774	8.13	0.030
IT Operating Expense							
<$1 M	163	ref	ref	ref	ref	ref	ref
≥ $1 M	428	8.13	0.002	0.22	0.929	6.67	0.100
IT Capital Expense							
<$75,000	193	ref	ref	ref	ref	ref	ref
≥ $75,000	313	6.24	0.023	-1.71	0.518	7.86	0.048
Total IT Staff							
<10	248	ref	ref	ref	ref	ref	ref
≥ 10	318	8.34	0.007	1.21	0.653	5.56	0.297
							
		Dec. Support		Effectiveness		User Support	
Teaching Status							
Non-teaching	474	ref	ref	ref	ref	ref	ref
Teaching	141	4.21	0.017	4.03	0.075	8.84	0.002
Urban Safety Net Status							
Non-safety net	535	ref	ref	ref	ref	ref	ref
Urban safety net	80	2.96	0.488	-6.71	0.206	2.58	0.341
Bedsize							
<350	268	ref	ref	ref	ref	ref	ref
≥ 350	347	2.50	0.081	1.01	0.570	4.00	0.072
Ownership							
Not-for-profit	347	ref	ref	ref	ref	ref	ref
Government	80	3.93	0.288	-0.13	0.971	-1.45	0.694
For-profit	170	-0.37	0.794	1.04	0.631	0.67	0.805
Average Age of Plant							
< 10 years	367	ref	ref	ref	ref	ref	ref
≥ 10 years	248	-1.39	0.398	-0.45	0.791	-2.90	0.273
Total Margin							
<0.02	236	ref	ref	ref	ref	ref	ref
≥ 0.02	379	0.77	0.624	2.62	0.167	1.42	0.572
IT Operating Expense							
<$1 M	163	ref	ref	ref	ref	ref	ref
≥ $1 M	428	2.42	0.203	0.85	0.690	0.31	0.919
IT Capital Expense							
<$75,000	193	ref	ref	ref	ref	ref	ref
≥ $75,000	313	2.46	0.069	-0.10	0.957	-1.62	0.540
Total IT Staff							
<10	248	ref	ref	ref	ref	ref	ref
≥ 10	318	4.23	0.083	1.41	0.606	1.43	0.727

* The unadjusted and adjusted regression models were adjusted for percentage of complete responses and clustered by hospital. The adjusted models include coefficients adjusted for bed size, total margin, and ownership. ref-reference group.

In the adjusted multivariable model, several of these associations persisted (Tables 2 and 3). Teaching hospitals had higher CIT scores (4.6 points higher, p = 0.002) than non-teaching hospitals. Hospitals with higher IT operations expenses, capital expenses, and larger IT staff continued to have higher automation scores (p < 0.05 in all cases). In contrast, hospitals with larger bed size or higher total margins did not have higher CIT, automation, or usability scores in the adjusted models. In addition, adjusted scores for urban safety net hospitals were not lower than those for non-safety net hospitals in any category. In the adjusted analysis, the type of ownership (church or not-for-profit, government, or for-profit) was not related to CIT, automation, or usability scores.

The adjusted analyses were repeated for each of the automation and usability sub-domains. Automation of test results were statistically significantly higher for teaching and not for profit hospitals and hospitals with larger bed size, greater total margin and IT capital expenses (p < 0.05 in all cases). Teaching hospitals also scored more highly on the decision support and user support sub-domains (p < 0.05 in both cases). Hospitals with a lower average age of plant (<10 years), and larger IT operating expenses, IT capital expenses, and IT staff had higher order entry scores (p < 0.05 in all cases).

In a separate sensitivity analysis, we divided each of the IT spending variables (IT operating expense, IT capital expense, and IT staff) by bed size to normalize these variables for the organization's size. We found no relationship between the normalized IT expenditure variables and CITAT scores, indicating that positive associations in the original analysis (in particular, higher automation scores associated with higher IT expenditures) diminished after accounting for bed size.

### Relationship between Automation and Usability

**Figure 2:**
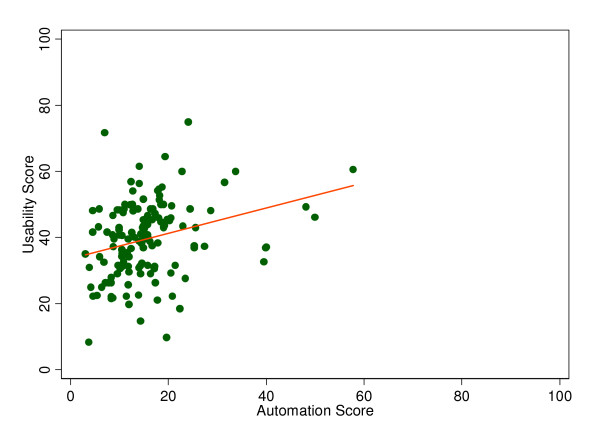
Scatter plot displaying the relationship between automation and usability scores by hospital.

Every 10-point increase in the automation score was associated with a usability score 3.8 points higher (Figure 2, p < 0.01). The magnitude and significance of this relationship held after adjustment for bed size, total margin, and ownership status (3.5 points, p < 0.01).

## Discussion

Many studies evaluating the adoption of CIT consider implementation as a binary event; in other words a technology such as computerized provider order entry (CPOE) is introduced into a group of hospitals and these hospitals are then compared to hospitals without CPOE. This approach makes it difficult to generalize results because technology implementations are often on-going processes with no distinct end point. The same CPOE system is likely to have different performance characteristics at 2 years post-implementation compared to 6 months post-implementation, partly as a result of dynamic changes involving both the technologic and organizational processes. Furthermore, the definition of information technology is rarely standardized from the perspective of the respondent; what may be defined as CPOE at one institution may be significantly different in scope, maturation, capability, and performance characteristics at another institution. It would be challenging to simply apply results based on simple terminologies cross-sectionally to different hospitals. The Clinical Information Technology Assessment Tool (CITAT) examines information technology capabilities in the hospital within the context of the *socio-technical environment *of the organization. This view holds that successful IT implementations jointly optimize both the technology and the social aspects of an organization, and that one aspect cannot be understood without knowledge of the other[12,16] The CITAT was designed with these concepts in mind and avoids simple terminological definitions of hospital IT that may not account for the usage, maturation, and capabilities of the information system and the organizational context in which it operates. Instead, the CITAT asks physicians whether a host of specific clinical activities in the hospital are routinely and preferentially conducted using computers. If there is insufficient user training, if the technology itself is unfriendly, or if the physician and organizational routines are not aligned with the technology, the CITAT scores for that hospital will be low, regardless of the cost or scope of the technologic acquisition. This approach allows the IT variable to be standardized across study hospitals and renders highest scores to those hospitals in which the technology, organizational routines, and clinical users are self-reinforcing, a fundamental feature of a highly optimized socio-technical environment [12,17].

In exploring which hospital characteristics are most associated with highly automated and usable clinical information systems as measured by the CITAT, we found that hospitals with larger information technology staff, budgets, and capital expenses had statistically significantly higher scores on automation, test results, and order entry scores. Spending on these factors alone appears to be more relevant than other structural factors, such as bed size, ownership status, and total margin, and persisted after adjustment for these factors. In a separate sensitivity analysis, however, after we normalized each of these factors for hospital size the association diminished or disappeared. Although bed size, by itself, was not related to higher automation scores, these results suggest that larger hospitals may enjoy an economy of scale with respect to the high fixed costs associated with large IT projects. Achieving this level of cost-effectiveness with respect to IT spending may be more challenging for smaller hospitals. Likewise, teaching hospitals, perhaps because of their history of innovation and experimentation, appear to embrace information technologies sooner than other types of hospitals. These hospitals scored higher on the CIT score and on multiple automation and usability sub-domains. As with other innovations in medicine, it is possible that academic physicians advocate for newer information technologies, increasing the speed of its adoption in these organizations.

Contrary to our expectations, a number of hospital characteristics do not appear to be related to the CITAT scores. Ownership status is not significantly associated with any of the IT variables, with the notable exception of test results. Within this latter sub-domain, not-for-profit hospitals scored 20 points higher than for-profit hospitals. Historically, test results has been among the earliest components of the information system to be automated and it is possible that not-for-profit hospitals, which constitute the more traditional form of hospital organization, may have more experience developing this component of their information systems [18]. Though there has been significant attention placed on the promise of computerized order entry systems to reduce medical errors, starting with the IOM reports in the 1990s, fewer hospitals have successfully installed such systems. We found that hospitals with older age of plant (i.e., building) scored 8 points lower on the order entry sub-domain. One might suspect that newer hospital facilities would be more easily equipped with computerized order entry systems than hospitals with older physical facilities, as these results suggest. Perhaps more important than the age of the building is the newness of its technological infra-structure. The latter may not necessarily correlate with the building age, though it could be captured in the age of plant variable and may explain the findings we observe.

Historically, urban safety net hospitals in the United States are least able to meet the challenges associated with acquiring new medical technology [19]. These hospitals balance multiple claims on their resources, perhaps reducing the capability to invest in the information technologies that support healthcare. Our analysis suggests, however, that urban safety net hospitals in Texas do not significantly trail their peers. Due to their size and scale, these hospitals may achieve IT parity because they can afford the fixed costs necessary for the IT infra-structure and have decided to pursue this course. In addition, all of the safety net hospitals in this sample are major teaching hospitals. Thus, it is difficult to differentiate between the effects of teaching status and safety net status.

According to recent estimates, adoption of clinical information technologies remains low but follows certain patterns [18,20]. Our findings are consistent with these trends. Historically, the computerized display of lab results has been among the first aspects to be automated [20]. In the last decade, digitization of radiological images has also increased [20]. Both of these components fall under the test results sub-domain, which in our study showed the greatest degree of adoption. Though some hospitals may be experimenting with computerized order entry and decision support, these efforts have not yet translated into systems that physicians widely use, as indicated by the low scores in these areas. Electronic decision support is perhaps the most challenging component to implement since it requires all other components first. In this study, notes & records scores were higher than scores for order entry and decision support, consistent with this theory and other studies [18].

Usability items in the CITAT do not presuppose the use of technology. The usability domain is constructed to measure the ease, effectiveness, and support of the information system regardless of the technologies in place [13]. As an example of the types of questions in this domain, one of the survey items asks whether physicians are able to obtain adequate computer support in less than 2 minutes. As might be expected, we found that usability scores were generally higher than automation scores. It is feasible that thoughtfully planned paper-based systems could produce usability scores higher than, or equal to, systems which employ poorly designed electronic processes. However, consistent with two previous studies, we found that a higher automation score correlated with higher usability scores, suggesting that digitization may be necessary to produce usable information systems. Alternatively, these results may indicate that physicians' expectations are changing; electronic processes may be perceived to be more usable than non-electronic processes, independent of overall merits, and therefore are rated more highly. Usability of the information system, an often elusive goal for hospital systems, was not specifically associated with any of the hospital characteristics we measured, with the exception of teaching status. In that case, hospitals with a teaching affiliation had higher user support scores than non-teaching hospitals. Our results suggest that usability may be more dependent on factors we did not measure as part of our set of hospital characteristics; these may include the quality and direction of leadership at the institution, the focus on quality improvement, and the concentration on human factors engineering in designing the information system. This will need to be further examined in future studies.

This study has important limitations. Our analysis explores a number of hospital characteristics, raising issues of multiple testing and increasing the probability of some false-positive relationships. As with all cross-sectional studies, positive associations will need to be confirmed in repeated studies. A Bonferroni correction for the number of tests performed would have eliminated many of the significant relationships we report. However, the Bonferroni method of correction for multiple testing is itself controversial, and argued by some to be too severe a method for correction [21]. The purpose of this study was to find potential relationships to explore further, given that the explanatory power of a cross-sectional study may be weak despite the construction of a well-validated instrument. Appropriate assessment of information technology requires multiple methods. Survey-based methods are one important method, but other methods such as electronic queries, time-motion studies, and qualitative analyses are needed to arrive at a complete portrait of an information system. Furthermore this study attaches importance to higher scores on the CITAT, as a measure of the strength of the socio-technical environment at the hospital. However, we do not yet know whether, and to what degree, CITAT scores correlate with important clinical and financial outcomes. These relationships will need to be assessed in the future.

## Conclusion

This study explores the relationship between hospital characteristics and information system characteristics among a diverse set of urban hospitals in the United States. Our findings suggest that those hospitals with an academic affiliation or those who spend significantly on IT capital and staff achieve higher automation scores. We found that fewer of the hospital characteristics we measured were meaningfully associated with usability scores. Further studies, using a variety of methods, should examine what organizational factors, such as policies, norms, and cultures, could explain these relationships.

## Competing interests

The authors declare that they have no competing interests.

## Authors' contributions

RA and NRP conceived of and designed the study. RA and ACC disseminated the survey and acquired the data. RA, NRP, LP, MDW, DJG participated in the analysis and interpretation of the data. RA, NRP, ACC drafted the manuscript; RA, NRP, LP, MDW, DJG made critical revisions to the manuscript; All authors participated in the statistical analysis and read and gave final approval to the manuscript. Both RA and NRP had full access to all of the data in the study and take responsibility for the integrity of the data and the accuracy of the data analysis. None of the authors have any conflicts of interest, including specific financial interests and relationships and affiliations relevant to the subject matter or materials discussed in the manuscript.

## Pre-publication history

The pre-publication history for this paper can be accessed here:


